# A Systematic Review of Observational Dyadic Persons Living With Dementia: Caregiver Communication Instruments

**DOI:** 10.1093/geroni/igaa057.900

**Published:** 2020-12-16

**Authors:** Sohyun Kim

**Affiliations:** University of Iowa, Iowa City, Iowa, United States

## Abstract

To facilitate social interaction and providing quality care in persons living with dementia (PLWD), an effective means of evaluating communication quality between PLWD and their caregiver is needed. However, there is no systematic review of current instruments to assess dyadic PLWD-caregiver interactions in various care settings. The purpose of this review was to critically evaluate existing dyadic observational communication instruments used to provide recommendations. A systematic review using the Preferred Reporting Items for Systematic Reviews and Meta-Analyses Guideline was conducted. Literature that were published by May 2019 in English were searched from CINAHL, AgeLine, PsychINFO, Communication and Mass Media Complete, ProQuest Dissertations and Theses Global, and Scopus. Keywords were “communication strategy,” “communication,” “caregivers,” “dementia,” and any combination of these terms or MeSH terms. Data were extracted including development process, operational concept, target population and setting, items/scoring format, psychometric properties, and research/clinical use. A total of 3042 articles were identified and 15 instruments from 29 studies were evaluated by the scoring of 12 psychometrics: participants/items ratio, reliability (internal consistency, intra-rater, inter-rater), and validity (content, concurrent, predictive, known group difference, divergent/discriminant, convergent, structural). The total score was ranged from 0 to 22 (high quality: 16-22, moderate: 8-15, low: 0-7). There was no instrument with high quality assessing dyadic interaction. Only one instrument was evaluated as moderate quality (modified Behavioral Observation Scoring System, BOSS). While existing instruments are still in the early stages of development and testing, they demonstrate potential evidence that may require further testing before application in research and clinical practice.

